# Eye Tracking in Virtual Reality: a Broad Review of Applications and Challenges

**DOI:** 10.1007/s10055-022-00738-z

**Published:** 2023-01-18

**Authors:** Isayas Berhe Adhanom, Paul MacNeilage, Eelke Folmer

**Affiliations:** 1 University of Nevada Reno, 1664 N Virginia St, Reno, NV 89557, USA

**Keywords:** Eye tracking, Virtual reality

## Abstract

Eye tracking is becoming increasingly available in head-mounted virtual reality displays with various headsets with integrated eye trackers already commercially available. The applications of eye tracking in virtual reality are highly diversified and span multiple disciplines. As a result, the number of peer-reviewed publications that study eye tracking applications has surged in recent years. We performed a broad review to comprehensively search academic literature databases with the aim of assessing the extent of published research dealing with applications of eye tracking in virtual reality, and highlighting challenges, limitations and areas for future research.

## 1 Introduction

Catering the stimulus to the user’s actions, such as head movement, eye movement, and hand movement, is the core principle of virtual reality (VR). Head-mounted display (HMD)-based VR depends on the ability to track head movements and render visual scene motion contingent on head movement. This advance was made possible by improvements in head tracking technology. Moving forward, similar improvements in HMD-based eye tracking technology will allow for fundamental advances in VR applications based on eye movement.

Consumer VR HMDs have significantly advanced in recent years in terms of tracking, latency, refresh rate, resolution and optics (Koulieris et al. 2019) with major consumer platforms (HTC Vive, Oculus Rift, Sony VR) already having presented second or third generation HMDs. Eye tracking technology has been commercially available for decades on desktop displays, but in recent years, a number of commercially available solutions have been developed that facilitate eye tracking on consumer VR HMDs. As a result, research and development surrounding eye tracking in HMDs has accelerated and expanded in recent years.

A number of literature reviews have provided an overview of general eye tracking applications (Duchowski 2002) and gaze-based interaction (Duchowski 2018) without focusing on VR. Additionally, there are several reviews, that we highlight in this review, that focus on specific applications of eye tracking in VR, including reviews by Rappa et al. (2019), Souchet et al. (2021), Lutz et al. (2017), Harris et al. (2019), and Souchet et al. (2021). However, to the best of our knowledge, no reviews exist that exclusively aim to provide a broad overview of eye tracking applications for VR HMDs. We believe that interest in this area is growing rapidly, given the large number of studies that utilize eye tracking in a VR setting. It is therefore timely to provide a broad overview of the applications, challenges and limitations of eye tracking in VR.

This paper provides a broad review of current and seminal literature of applications of eye tracking in VR and identifies limitations, challenges and areas for future research. The rest of this paper is organized as follows: Sect. 2 discusses background concepts about eye movements and eye movement tracking methods in VR that will help the reader to understand the concepts discussed in later sections. Section 3 presents the applications of eye tracking in VR by organizing them into seven broad application areas. Finally, Sect. 4 discusses the current challenges and limitations faced by eye tracking in VR.

## 2 Background concepts

### 2.1 Types of eye movements

There are several distinct types of eye movements that serve different functions and are controlled by distinct neural circuits. Optimization of eye tracking for VR applications often requires identifying and distinguishing these different eye movement types and exploiting knowledge of visual and cognitive processing during eye movements to achieve the desired outcome(Holmqvist et al. 2011).

The need for eye movements arises because human visual acuity is not uniform across the visual field. Visual acuity is highest at the fovea, a central region of the retina(Cowey and Rolls 1974). The foveal region has the highest density of photoreceptors that allows parts of the visual field that fall on it to be seen with the highest detail (Curcio et al. 1990). Outside the foveal region, visual acuity drops gradually towards the edges of the retina. Below we will describe several eye movement types and their relevance including: saccades that just move the eyes from target to target, smooth pursuit that allow tracking moving objects, reflexive eye movements that stabilize vision, and vergence eye movements that coordinate the two eyes to allow binocular fixation at different depths. In the rest of this section, we describe these eye movements and their functional role in greater detail.

The subjective experience of being able to see the entire visual field in high acuity is achieved by scanning the visual field with successive eye movements known as saccades and integrating the high acuity snapshots of the scene that are obtained, a process known as trans-saccadic integration (Melcher and Colby 2008). Saccadic velocities may reach as high as 400 to 600 deg/sec, with durations lasting from 30 to 120 msec, and amplitudes in the range 1 to 45 degrees; there is a systematic relationship between amplitude, duration, and velocity known as the saccadic main sequence (Leigh and Zee 2015). Under natural circumstances, gaze shifts of greater than about 30 degrees are typically achieved by a combination of saccade and head movement. Vision is typically blurred during a saccade which allows for manipulation of the visual scene to go largely unnoticed during the saccadic interval.

Between saccades, the eyes typically remain relatively still for an average duration of 200-300 ms to fixate on objects of interest in the scene. Analysis of fixation locations provides information about the user’s attentional state and the scene content that the user is interested in. However, the eyes are never completely still during fixations, because there are miniature fixational eye movements that occur during this time of relative stillness. Fixational drifts are small and slow movements away from a fixation point (Otero-Millan et al. 2014). Drifts are often followed by small faster movements known as microsaccades that bring the eyes back to the fixation point; these small movements are typically less than 1 degree (Otero-Millan et al. 2014).

Pursuit eye movements occur when the eyes track a slowly moving visual target in order to stabilize the image of that target on the retina. Targets with velocities of greater than 30 deg/sec cannot be effectively tracked by pursuit eye movements, but catch-up saccades are triggered to compensate for this lag. Most people are unable to initiate pursuit without a moving visual signal. Nevertheless, pursuit is considered to be a voluntary eye movement (Robinson 1965).

Another type of eye movement that serves to stabilize the image on the retina is known as the vestibulo-ocular reflex (VOR). These movements act to rotate the eyes to null foveal retinal image motion that would result from head movement relative to the stationary environment. The VOR is driven predominantly by vestibular signals that transduce inertial cues to head motion, both linear and angular (Ramaioli et al. 2019). Vestibular-driven movements are supplemented by visually driven movements that respond to uncompensated image motion at the fovea known as retinal slip. The resulting stabilizing eye movement, referred to collectively as the visually enhanced VOR, allows us to see the world as clear and stable despite head movement (Leigh and Zee 2015).

Vergence eye movements are the simultaneous movements of both eyes in opposite directions that allow the two eyes to change binocular fixation distance. The nearer the target, the greater the convergence that is required. Thus, the vergence angle of the eyes along with disparity in the images presented to the left and right eyes determines how users perceive stereoscopic depth. Because vergence is associated with target depth, there is a linkage between neural signals that drive vergence and those that drive accommodation, or focal state, of the lens of the eye, which also needs to vary with target depth to ensure that the retinal image is in focus (Toates 1974).

Several of these eye movement types can occur simultaneously, such that the total movement of the eye is the result of superposition of contributing movements, making it difficult to identify and quantify distinct types of eye movements. In addition, eye movements measured in head coordinates can be ambiguous and difficult to characterize without information about scene content and head movement. Inaccurate and/or imprecise classification and quantification in turn compromises the use of eye movement data for VR applications. Advanced identification and classification methods that make use of all available information are therefore an active area of research (Kothari et al. 2020).

### 2.2 Eye tracking methods in VR HMDs

Three methods have been used to track eye movements in HMDs: (1) electro-oculography (EOG), (2) scleral search coils, and the most common, (3) video oculography (VOG).

EOG measures the orientation of the eye by placing electrodes on the skin around the eye which measure the resting potential of the eye. The electrodes can be easily incorporated into the HMD where it contacts the face (Bulling et al. 2009; Shimizu and Chernyshov 2016; Xiao et al. 2019; Kumar and Sharma 2016). The method works because the eye is a dipole that is positively charged toward the cornea and negatively charged toward the retina. The difference in voltage across electrodes placed on opposite sides of the eye (e.g., left and right) map well to eye orientation (e.g., horizontal position of the eye) (Mowrer et al. 1935). One drawback is that EOG provides a fairly imprecise measure of eye position, but EOG is the only method that allows tracking while the eyes are closed.

**Fig. 1:**
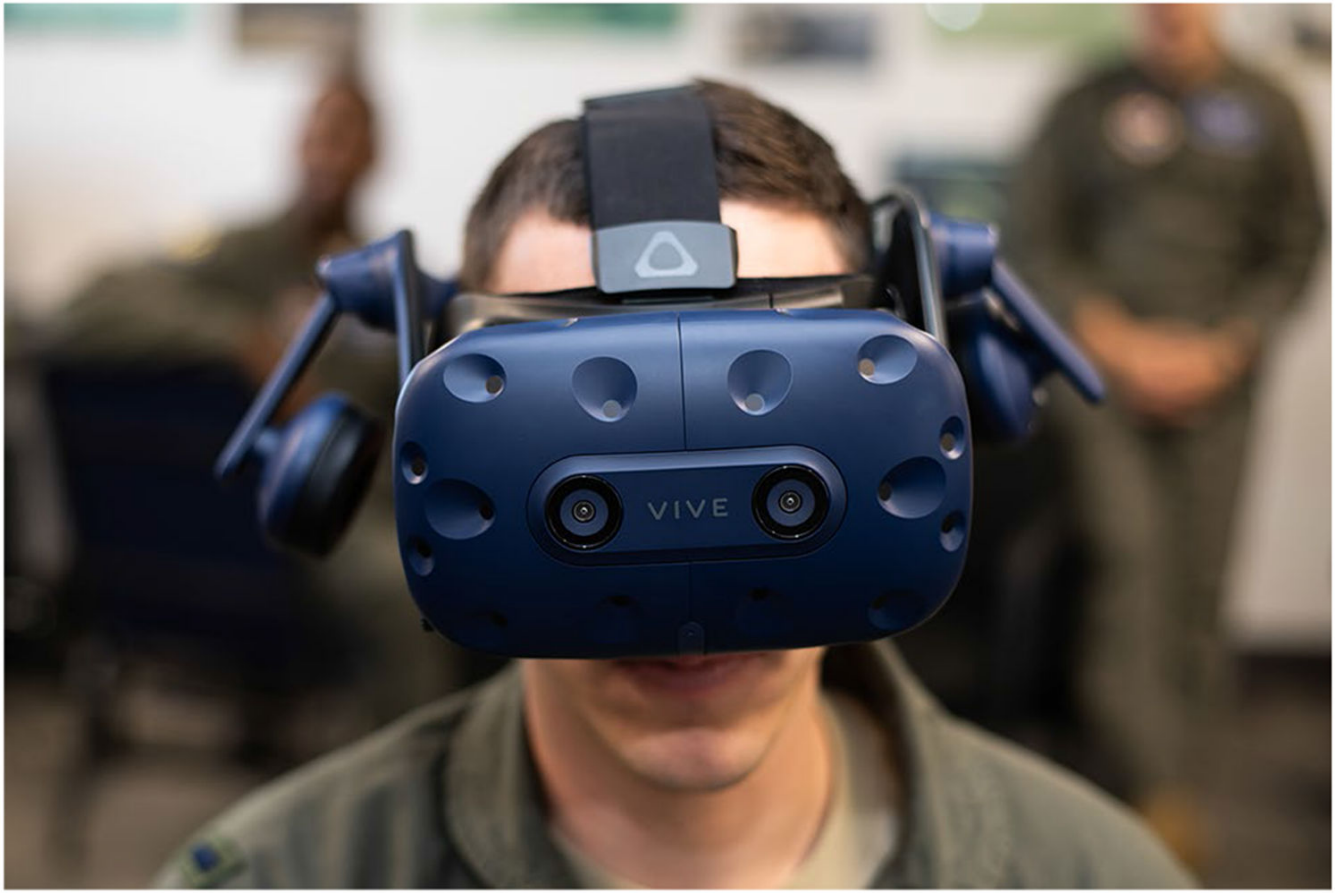
An HTC Vive Pro Eye HR HMD with integrated VOG-based eye trackers (left). A VR HMD with scleral search coil-based eye tracking system (right). Image courtesy of Whitmire et al. (2016)

The scleral search coil method works by tracking the orientation of a wire loop that is embedded in a contact lens worn by the user (Robinson 1963). The user’s head is positioned between Helmholtz coils which generate a uniform magnetic field. When the eyes move in the uniform and known magnetic field, electric current is induced in the scleral coils indicating the horizontal, vertical and torsional orientation of the eye. This method is highly accurate and precise with reported spatial resolution of less than 0.1° and temporal resolution that is greater than 1KHz (Whitmire et al. 2016). However, this method is challenging to implement in HMDs not only because of the need for wired contact lenses but also because the Helmholtz coils must be head-mounted and consequently the resulting magnetic field is much smaller in volume. Nevertheless, an HMD with scleral coil system (see Figure 1) has been developed Whitmire et al. (2016) for use in specialized cases where high-precision tracking in an HMD is required, for example to validate alternative tracking systems.

By far, the most common eye tracking method used in HMDs is VOG (Duchowski 2007). Currently commercially available HMD-based eye trackers including Tobii, Pupil Labs, Varjo, and Fove employ VOG-based eye tracking. Images of the eyes are captured by cameras mounted in the HMD and analysis of the video frames indicates eye orientation. Most often, analysis methods rely on identification of the pupil and perhaps other landmarks to recover eye orientation. There are many analysis methods for recovering eye position from eye images, and methods implemented by commercially available systems are often proprietary (Holmqvist et al. 2012). This is an active area of research, but in-depth discussion of eye image analysis methods is beyond the scope of the present paper. Regardless of the method, better quality eye images translate into better quality tracking. Camera position is an important factor limiting image quality. Systems that film the eye front-on through the HMD optics using a hot mirror typically perform better than systems that mount the cameras obliquely thereby bypassing the HMD optics (Richter et al. 2019). Spatial and temporal resolution of the eye cameras also limit tracking performance. The current standard in commercially available systems varies. For example, the Pupil Labs VR/AR plugin system has 192x192 spatial resolution at 200 Hz, while the Varjo system has 1280x800 spatial resolution at 100Hz.

### 2.3 Technical aspects of eye movement tracking

#### 2.3.1 Eye tracking data quality

High quality eye tracking data are an important prerequisite for any application that uses eye movement data. Eye tracking data quality refers to the performance of an eye tracker as measured by various metrics including spatial precision, spatial accuracy, sampling rate, and latency.

*Spatial Precision* is related to the ability of an eye tracker to reproduce measurements accurately over time. Spatial precision of an eye tracker is commonly measured by calculating the sample-to-sample root-mean-square angular displacement (RMS) and the standard deviation of samples collected within a given time window. The amount of precision needed for an eye tracking-based application depends on the type of the eye movement that needs to be recorded and the type of eye tracking task. Applications that use small fixational eye movements, like tremors, drifts and microsaccades require high-precision eye trackers. Andersson et al. (2010) point out that for such tasks the eye tracker should have an RMS precision value that is lower than 0.03°.

*Spatial Accuracy* is the average angular deviation between the true gaze position and the gaze position estimated by an eye tracker (Holmqvist et al. 2012). Spatial accuracy of an eye tracker is measured as the distance, in degrees, between the positions of a target point and the average position of a set data samples collected from the eye tracker within a given time window. Lack of accuracy may not pose any problems when the areas of interest in an application are large and are distant from each other. However, in applications with closely spaced stimuli, small inaccuracies could be critical, and could lead to the information obtained from the user or the action executed by the user being different from what the user intended.

*Latency* refers to the delay between eye movement events and the time these events are received by our system. There are various factors that contribute to data latency in the eye movement tracking pipeline including the sampling rate, the time to compute the gaze position from the eye images, the time to filter and post-process the data and the time to transmit the data to our VR display. Latency could cause elements of the stimulus that are supposed to respond to eye movement to lag behind the eye movements. When this lag is big enough that it is perceptible by the user, it could severely affect the virtual experience. This could be particularly problematic in interactive applications or gaze-contingent displays which change some parts of the stimulus in real time in response to eye movements.

*Sampling Rate* or sampling frequency of an eye tracker refers to how many times per second the eye is recorded by the eye tracker. Sampling rate determines the type of events that can be measured with your eye trackers and the suitability of the eye tracking data for a particular application. For example, the precise measurement of small amplitude eye movements such as fixational eye movements and low amplitude saccades requires eye trackers with high sampling rates. Andersson et al. (2010) argue that based on the Whittaker–Nyquist–Shannon theorem Shannon (1949), the sampling rate should at least be at least twice the speed of the eye movement to be recorded.

#### 2.3.2 Calibration

Calibration is the process by which the eye tracking system finds a mapping function that maps coordinates reported by the eye tracker to the coordinates of a gaze point in the visual environment (Harezlak et al. 2014). Most HMD-based eye trackers use a standard point-based calibration procedure. The procedure successively shows a set (usually 5 to 16) of small point targets to the user and the user is asked to fixate on each target for a few seconds. The calibration procedure requires willful engagement and cooperation from the user to be successful, because the mapping function requires the user to fixate on each target for the full duration that the target is shown on the environment. Due to differences in eye attributes and the geometry of the eye tracking setup, most eye tracking use-cases require users to calibrate the eye tracking system before they can use it. However, some use-cases, such as smooth pursuit-based interaction, just employ the user’s relative eye motion in reaction to scene objects; therefore, they do not need to calibrate the eye tracking system Zeng et al. (2020).

## 3 Eye tracking applications in VR

To improve readability, the presentation of the review is organized into seven broad eye tracking application areas: display and rendering; user interaction; collaborative virtual environments; education, training and usability; security and privacy; marketing and consumer experience research; and clinical applications.

### 3.1 Display and rendering

Knowing where the user is looking can provide advantages in both functionality and efficiency of rendering in VR. Building truly immersive VR experiences would require the development of HMDs that are capable of rendering content with visual quality that is close to that of the human visual system. To enable such display systems, it is estimated that we would need to deliver more than 100 Gb/sec data to the HMD (Bastani et al. 2017). Achieving this data rate on VR systems is challenging. However, due to the physiological limitations of the eyes, the human visual system can only consume a small percentage of this data stream. The human eye has very high acuity in the central 5.2° region—the fovea—of the retina. Outside this region, visual acuity falls off as we move toward the periphery of the retina. The fovea covers around 4% of pixels on consumer VR systems (Patney et al. 2016) with about 96% of the rendered pixels in HMDs falling in regions of the retina that have low visual acuity. Gaze-contingent (foveated) display systems (GCDs) exploit this phenomena to increase performance and reduce computing costs in HMDs by rendering content that falls outside the fovea with low resolution or level of detail (Guenter et al. 2012). When coupled with high quality eye tracking, GCDs could help future HMDs to have a wide field-of-view with higher resolutions and refresh rates. On top of reducing the computational requirements and improving speed in VR systems, GCDs also have other highly relevant advantages for VR that include reducing streaming content bandwidth requirements, reducing visual discomfort in VR and alleviating VR sickness.

#### 3.1.1 Improving rendering efficiency

Foveated Rendering renders the areas of the display that lie at the user’s center of eye gaze with the highest resolution and degrades the resolution with increasing eccentricity (Patney et al. 2016; Guenter et al. 2012). This leads to improvements in rendering performance and rendering quality, and has been shown to achieve up to 50-70% in performance savings (Weier et al. 2017). Popular implementations of foveated rendering include those of Guenter et al. (2012) and Patney et al. (2016).

Compression of the peripheral parts of the scene, however, could introduce various perceptible artifacts such as tunnel vision, aliasing and flicker that could distract users and reduce immersion (Patney et al. 2016). To address these artifacts, Guenter et al. (2012) used three gaze centered concentric circles with resolution degrading progressively towards the periphery. Whereas Turner et al. (2018) propose a technique to reduce motion induced flicker in the periphery using phase-alignment—aligning the rendered pixel grid to the virtual scene content during rasterization and upsampling.

#### 3.1.2 Reducing transmission load

Streaming immersive omnidirectional video (ODV), also known as 360° video, to VR devices is a growing trend. When ODV is viewed in VR, it allows the user to look around a scene from a central point of view and provides a more immersive visual experience than traditional 2D video playback. However, streaming ODV across content delivery networks and displaying it in a VR device are challenging in part due to the large resolution requirement of the video. Foveated rendering could be used to reduce computational rendering cost once the streaming content is available in the user device. However, streaming the content from where it is stored to the end user’s device is in itself a big challenge. As discussed above, viewers can only watch a small part of the streaming content due to the physiological constraints of the human eye. As a result, gaze-contingent (foveated) transmission techniques have been proposed to minimize the amount of data transmitted to the user’s device (Lungaro et al. 2018; Romero-Rondón et al. 2018; Ozcinar et al. 2019). These techniques generally aim to reduce the amount of data transferred to the user’s device using gaze-adaptive streaming. An exemplar gaze-adaptive streaming technique is that of Lungaro et al. (2018) where user’s eye gaze is continuously tracked and gaze positions are transmitted to a *Foveal Cloud Server*, which, in return, transmits the content with high visual quality around the users’ fixations points while lowering the bandwidth required to encode the content everywhere else. Evaluation of the technique showed that it could lower the bandwidth requirement for streaming VR content by up to 83%.

#### 3.1.3 Reducing discomfort due to depth conflicts

Conventional stereoscopic 3D near-eye displays, like those used in VR HMDs, create 3D sensation by showing each eye a distinct 2D image where each image is rendered with slight differences to create binocular disparity. Binocular disparity is a critical stimulus to vergence, which is a critical depth cue. However, the distance between the user’s eyes and the image is fixed by the location of the display screen. As a result, although the 3D imagery is displayed at various depths, the eyes are always focused at a single depth. Thus, the display does not depict correct retinal blur which leads to the inability of the eyes to focus or accommodate correctly, causing loss of accommodation—another critical depth cue. This mismatch is called vergence-accommodation conflict (VAC) and is a source of visual discomfort including eye strain, blurred vision and headaches (Kramida 2016; Shibata et al. 2011; Hoffman et al. 2008).

Matsuda et al. (2017) provide a review of several “accommodation-supporting” displays that have been proposed to address VAC, including varifocal displays, monovision displays, accommodation-invariant (EDOF) displays, multifocal displays, retinal scanning displays, light field displays and holographic displays. Out of these approaches, varifocal displays utilize eye tracking to mitigate VAC. Varifocal displays use eye tracking to actively track the vergence of the eyes and use a focusing element with a variable focus to match the eye’s vergence.

Ocular parallax is another important depth cue that current stereoscopic displays fail to render accurately (Krajancich et al. 2020). Ocular parallax is a depth cue caused due to the centers of projection and rotation not being the same in the human eye, thus, leading to the formation of small amounts of depth-dependent image shifts on our retina when we move our gaze (Kudo and Ohnishi 1998). Although ocular parallax is an important depth cue that could significantly improve the impression of realistic depth when viewing a 3D scene, conventional stereoscopic rendering techniques, that are widely used on VR systems, assume that the centers of projection and rotation are the same and do not render ocular parallax. Konrad et al. (2020) introduced a gaze-contingent ocular parallax rendering technique that tracks the user’s eye gaze to render ocular parallax. The authors report that the technique improved perceptual realism and depth perception.

#### 3.1.4 Reducing VR sickness

The potential of gaze-contingent displays to reduce the incidence of VR sickness has also been explored. Adhanom et al. (2020b) explored the utility of foveated field-of-view (FOV) restriction to reduce VR sickness. FOV restriction (tunneling) is a popular technique to reduce visually induced motion sickness (Fernandes and Feiner 2016) that involves blocking the peripheral view of users by a restrictor to minimize optical flow in the peripheral parts of the retina which are sensitive to it. Most current implementations of FOV restriction do not respond to eye gaze and could reduce immersion and the sense of presence. Foveated FOV restriction (Adhanom et al. 2020b), however, implements a restrictor that moves with the user’s eye gaze that would allow the users to see a bigger part of the visual scene while still blocking their peripheral vision. This allows greater visual exploration of the environment when compared to fixed FOV restrictors.

#### 3.1.5 Summary

Current gaze-contingent display techniques have shown great potential to make rendering more efficient and improve presence and comfort in VR. However, it is still unclear how degradation of the peripheral image impacts attentional behavior and task performance in VR. Previous studies have shown that non-immersive gaze-contingent displays affect task performance (e.g., reading speed) negatively (Albert et al. 2019); therefore, further research is needed to understand the effect of GCDs on task performance in VR. Moreover, most of the eye tracking devices integrated in VR HMDs have high latency, lack precision and do not have simple calibration procedures. Latency could have a negative effect on gaze-contingent rendering by introducing perceptible delays to the rendering pipeline which could reduce immersion and cause discomfort in VR. Various approaches have been proposed to address latency including saccade end-point prediction (Arabadzhiyska et al. 2017), and machine learning-based gaze position prediction (Hu et al. 2019) techniques. However, there is still room for improvement and further research is needed to reduce latency in GCDs.

### 3.2 User interaction

Eye movement-based interaction interfaces employ realtime eye movements as a user interaction modality. Eye movement-based interaction could be particularly useful in situations where other interaction modalities are not preferred or available, for instance when the user has severe motor disabilities or when the user’s hands are occupied with other tasks. Although, using eye movements for interaction may not be as accurate as using hand-based controllers in VR, eye gaze can be much faster than conventional input devices (Špakov et al. 2014; Sibert and Jacob 2000). By eliminating or reducing the number of hand-based gestures in VR, eye gaze-based interaction has the potential to reduce the so-called gorilla arm syndrome—arm fatigue due to prolonged hand-based midair gestures (Boring et al. 2009; Cockburn et al. 2011)—which has been shown to limit the amount of time users spend in VR (Jang et al. 2017) . Similar to LaViola Jr et al. (2017), we classify the interactive applications into three categories: selection and manipulation, virtual locomotion and system control.

#### 3.2.1 Selection and manipulation

Manipulation, one of the fundamental tasks in both physical and virtual environments, refers to interaction tasks that involve selecting and manipulating virtual objects in a virtual environment (VE). These could be distilled into basic tasks that include pointing at, selecting, positioning, rotating and scaling virtual objects (LaViola Jr et al. 2017).

##### Selection

According to Bowman et al. (2001), a selection technique has to provide means to indicate an object, a mechanism to confirm its selection (confirmation of selection) and some form of feedback to guide the user during the selection task. Indication of an object can be accomplished through object touching, pointing, occlusion/framing or indirect selection. Eye gaze-based object indication is accomplished with pointing, whereas eye gaze-based confirmation of selection is accomplished through dwell and other bimodal mechanisms (Bowman et al. 2001).

Pointing is a fundamental interaction task that allows the user to point at objects or interaction elements they intend to interact with. Eye gaze-based pointing allows the user to select and interact with objects that are at a distance. The most common way of implementing eye gaze-based pointing in VR is to use the 3D gaze direction vector provided by the eye tracker, and to observe which objects in the scene intersect with the direction vector (Mohan et al. 2018; Sidenmark and Gellersen 2019; Cournia et al. 2003). Usually, a ray is cast based on the direction vector, and the first intractable object that the ray intersects with is considered to be the item that is being pointed at.

Various studies show that gaze-based pointing is faster than hand-based pointing as we are able to move our gaze faster to a target than our hands (Sidenmark and Gellersen 2019; Tanriverdi and Jacob 2000). However, due to inherent physiological characteristics of eye movements and technological limitations of eye tracking, eye gaze-based pointing is inaccurate compared to other common pointing interfaces such as hand- or head-based pointing (Špakov et al. 2014; Hansen et al. 2018; Qian and Teather 2017; Luro and Sundstedt 2019). The two main forms of inaccuracies in eye gaze-based pointing interfaces are caused by natural noise in eye tracking data and low eye tracking data quality. These issues are discussed in detail in Sect. 4.

Confirmation of Selection allows the user to confirm the selection of an object after they indicate it with a pointing interface. Selection with eye gaze alone is a relatively challenging task, necessitating the implementation of further mechanisms to enable selection when using eye-based interaction in VR. An added benefit of implementing other selection confirmation techniques is remedying the Midas touch problem (Jacob 1990)—an inherent problem for eye gaze only interaction techniques. The Midas touch problem arises from the difficulty of distinguishing between an intentional gaze interaction from natural eye movements—“everywhere you look, something is activated; you cannot look anywhere without issuing a command” (Jacob and Stellmach 2016).

Various techniques have been used to implement selection confirmation for gaze-based interaction in VR. Hansen et al. (2018) implemented a technique that uses eye gaze-based dwell for selection confirmation. Sidenmark and Gellersen (2019) implemented two head assisted techniques: Eye &Head Dwell, a confirmation technique where a dwell timer is only triggered by head-supported gaze shift but can be paused and resumed with eyes-only gaze; and Eye &Head Convergence, an alternative technique to dwell for fast target confirmation that allows users to confirm selection by aligning both the eye pointer and the head pointer over a target. Kumar and Sharma (2016) implemented a technique that uses blink or wink gestures for selection confirmation. Pfeuffer et al. (2017) allows users to point at objects with their eye gaze and select them with a pinch gesture with their hands. Pai et al. (2019) introduced a technique where users pointed at targets with their gaze and triggered actions using arm muscle contractions that were detected using electromyography. Qian and Teather (2017) used a keyboard button press for selection confirmation and eye gaze for pointing. Sidenmark et al. (2020) proposed a technique—Outline Pursuits—that utilizes smooth pursuits to allow users to select occluded objects in VEs.

##### Feedback

A selection technique should provide feedback to the user to give them a clear indication of the system’s status: Is the pointer following the eye gaze accurately? Has the system accurately recognized the intended target? and has the system selected the correct object (Majaranta and Bulling 2014)? As the eyes are sensitive to visual changes in the field of view, they instinctively try to shift attention to these visual changes. Thus, care should be taken when providing feedback to the user, as visually prominent feedback mechanisms could have unintended consequences of shifting the users gaze unless the feedback is provided on the selected object itself. Using non-visual feedback, such as auditory feedback, Boyer et al. (2017) would be an alternative approach. Examples of visual feedback for eye gaze-based interaction include: highlighting the selected object (Blattgerste et al. 2018); displaying an outline around the selected object (Sidenmark et al. 2020), and showing confirmation flags around the selected object (Mohan et al. 2018).

#### 3.2.2 Virtual locomotion (travel)

**Fig. 2:**
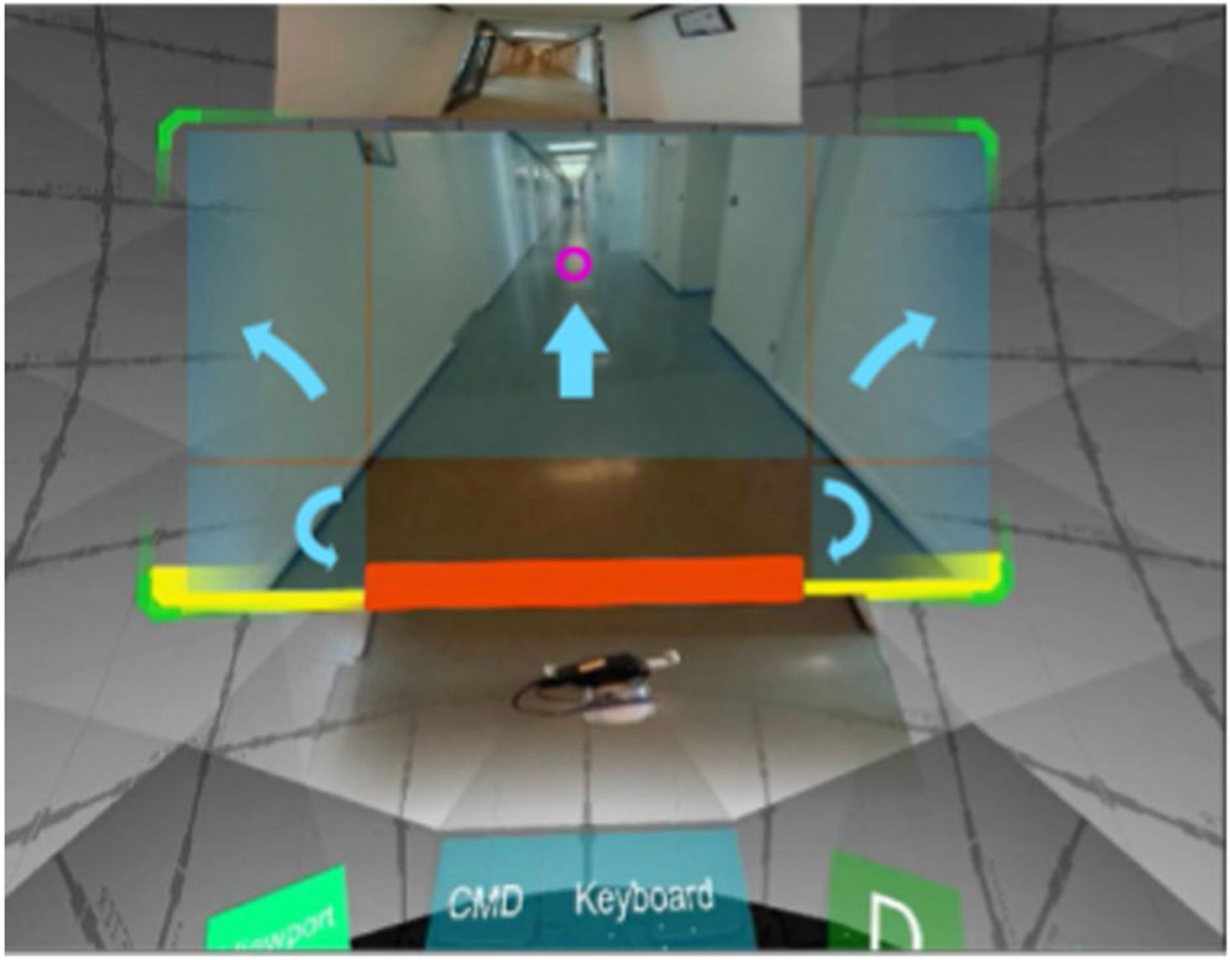
A gaze-interactive user interface overlayed over a virtual environment. Image courtesy of Zhang et al. (2019)

Virtual locomotion is the act of navigating VEs in VR. Designing efficient and universally accessible locomotion techniques presents considerable challenges (Al Zayer et al. 2020). Eye movements have been used to develop virtual locomotion interfaces that map eye movements to the control of the virtual viewpoint’s translation and orientation or to augment other navigation techniques Fig. 2.

Several implementations of eye movement-based virtual navigation use eye gaze-based steering interfaces, where 2D gaze interfaces are superimposed on the VE to allow users to issue steering commands with their gaze (Stellmach and Dachselt 2012). Zhang and Hansen (2019) proposed a steering-based virtual navigation technique that is used to train users who are disabled how to control wheeled tele-robots with their eye gaze. A similar implementation is that of Araujo et al. (2020), except that the proposed eye gaze-based virtual navigation technique is used to train people who are severely disabled on how to control wheelchairs in a safe simulated virtual environment. This review also included two other control interfaces in addition to overlayed steering user interfaces: continuous-control interface and semi-autonomous waypoint interface. In the continuous control interface, steering is implemented by measuring the depth and horizontal values of the gaze point intersection with the ground plane, and these measurements are used to calculate motor torque to drive the wheelchair. The semi-autonomous waypoint navigation allows the user to select waypoints (targets) in the ground plane using a dwell action; then, the application directs the motion of the user through the fastest route to the selected waypoint. The study found that the semi-autonomous waypoint-based method had superior performance compared to the two other techniques. Other implementations of eye gaze-based virtual navigation use point and fly techniques (Qian and Teather 2018; Zeleznik et al. 2005), where participants fly to a point in the VE by gazing at it. Eye tracking has also been used to implement orbital navigation techniques (Pai et al. 2017; Outram et al. 2018). Orbital navigation techniques—which are similar to fly-by camera shots in film and sports coverage—allow the user to move in an orbital path around points of interest. They maintain the point of interest in sight at all times and are particularly suited to observational tasks (Outram et al. 2018).

Eye tracking is also used to augment other popular navigation techniques like redirected walking. While real walking-based locomotion is the most natural way to travel in VEs, it is constrained by the size of the available physical tracking space. One interesting approach to overcome the limit of the physical space is using redirected walking techniques, whose primary goal is to allow the user to navigate VEs far greater than the physical tracking space. This is accomplished by manipulating the transformations of the user’s movements within the VE to give them the illusion of walking in straight paths in the VE while in reality they are walking in a curved path (Al Zayer et al. 2020). Ideally, the manipulations should be subtle enough to be imperceptible to the user. Techniques that detect the user’s eye movements to apply subtle translation of the user’s viewpoint during blinks (Nguyen and Kunz 2018) or saccades (Sun et al. 2018) are among the various approaches used to make translation changes imperceptible in redirected walking. Joshi and Poullis (2020) have proposed a technique that relies on inattentional blindness and foveated rendering to apply spatially varying transformations to different parts of the scene based on their importance in the field of view. Other eye movement-based approaches use the user’s eye gaze and probabilistic methods to predict the user’s future locomotion targets to reduce the number and magnitude of translation changes (Zank and Kunz 2016).

#### 3.2.3 System control

LaViola Jr et al. (2017) defines system control as an interaction task in which commands are issued to: (1) request the system to perform a particular function; (2) change the mode of interaction, or (3) change the system state. System control allows a user to control the flow of tasks in the system.

Perhaps the simplest form of eye gaze-based control mechanism is using blink as a binary input modality similar to a switch. Two example applications of blink-based control are the study by Xiao et al. (2019) that presented a technique where users can issue commands to the system, to control a VR-based music-on-demand system, by blinking in synchrony with a target button from several flashing buttons; and the study by Kumar and Sharma (2016) that proposed a technique where users could control a game using various blink commands including blink, double blink and wink eye movements. Requiring users to alter their natural blink rate, however, can cause eye strain, dry eyes and eye fatigue in users (Hirzle et al. 2020). Subjective results from the study by Kumar and Sharma (2016) also indicate that frequent blinking and winking leads to eye fatigue in users. Blink-based interfaces tend to be inaccurate because voluntary (intentional) blinks are hard to distinguish from natural blinks and thus require users to perform extended blinks. Extended blinks, however, have obvious disadvantages like slowing down the flow of interaction and blocking the user’s sight for the duration of the extended blink. Consequently, eye gaze-based system control applications mostly rely on the point-and-select paradigm discussed in Sect. 3.2.1.

Symbolic input—the input of characters and numbers—is an important and fundamental system control task. Symbolic input in VR remains to be challenging due to the fact that users’ eyes are obstructed from the physical world making the use of conventional text input devices, like physical keyboards, challenging, if not impossible. As a result, eye gaze has been explored as a potential symbolic input modality for VR. Gaze-based text entry in VR could be considered as a special form of target selection, where user’s use their gaze to select the keys of a virtual keyboard displayed in the VE. Rajanna and Hansen (2018) developed an eye gaze-based typing interface in VR and investigated how the keyboard design, selection method, and motion in the field of view may impact typing performance and user experience. The results of the study showed that gaze typing in VR is viable but constrained. Users’ perform best when the entire keyboard is within view compared to the larger than view keyboard (10.15 WPM vs 9.15 WPM), in addition the larger than view keyboard induces physical strain due to increased head movements. Ma et al. (2018) developed a hybrid gaze-based text entry system for VR by combining eye tracking and brain–computer interface (BCI) based on steady-state visual evoked potentials (SSVEP). The authors designed a 40-target virtual keyboard to elicit SSVEPs and to track gaze at the same time. The study compared the performance of the hybrid system to single-modality eye tracking-based and BCI-based techniques. The results indicate that the hybrid method has better performance than both the single-modality systems achieving a typing speed of around 10 WPM. It should be noted that gaze typing is very slow compared to conventional typing interfaces such as a conventional keyboard.

#### 3.2.4 Summary

Eye tracking has the potential to enable accessible and handsfree ways of input that require very little exertion (Majaranta and Bulling 2014). However, one technical challenge common to eye movement-based interaction applications is the rapid, accurate and precise identification of these eye movements that allows them to be used in real time as an input signal. Consequently, the exploration of new techniques that improve the quality of eye tracking data is an area that needs further research. With more sensors being embedded in VR HMDs, sensor fusion (i.e., combining eye tracking with other sensors) is another area for future research that holds promise to further increase the accuracy and robustness of using eye tracking for input. Eye tracking could be explored to enable individuals with severe motor impairments (i.e., quadriplegics or persons who are locked in) to navigate their avatar in VR. Though there might be significant challenges with using eye tracking for precise navigation, some sort of semi-autonomous approach could be used with a user providing broad navigation instructions. Of interest would be how this approach would affect VR sickness. Finally, our review reveals that there is scant literature on the use of eye-based input for manipulation tasks in VR.

### 3.3 Collaborative virtual environments

**Fig. 3:**
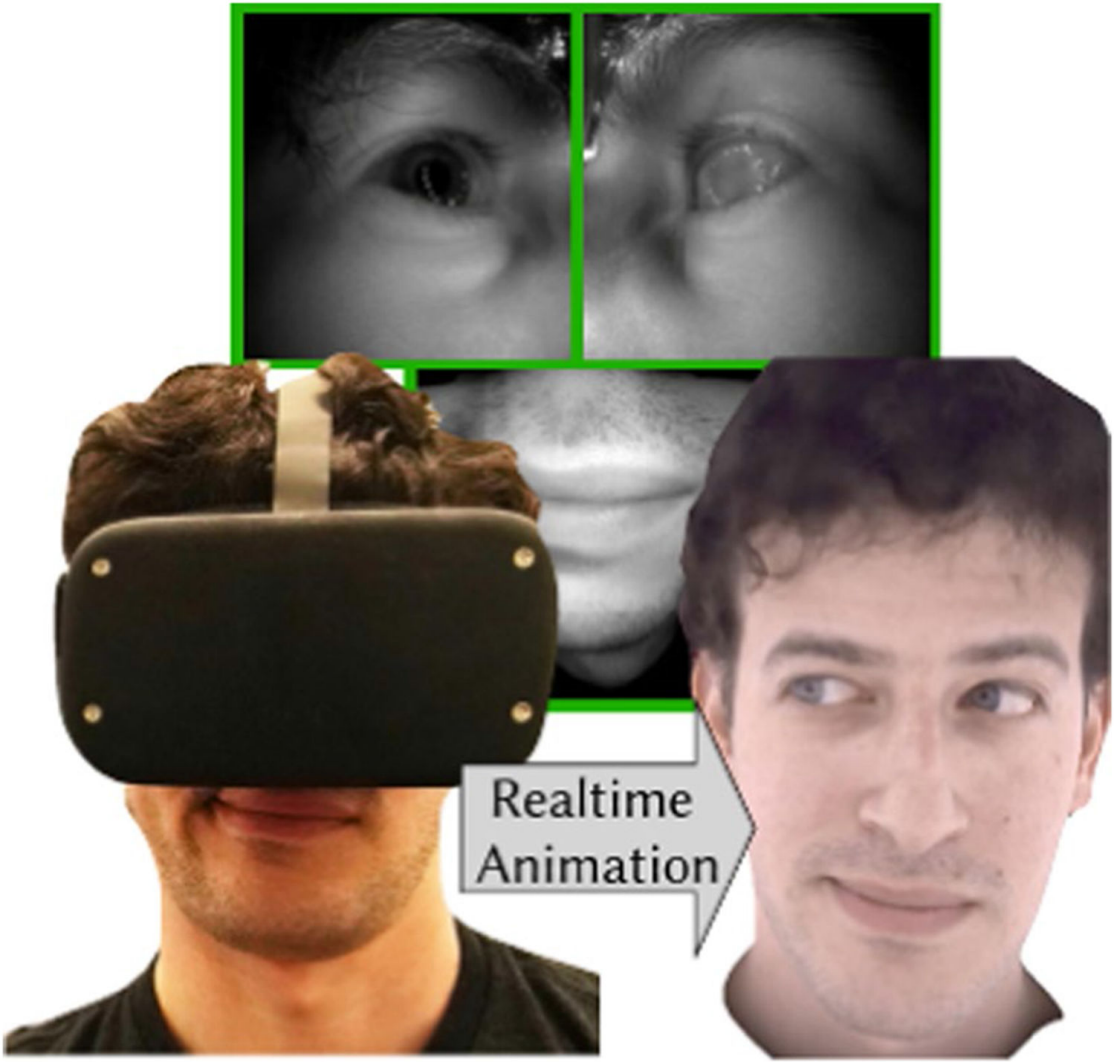
Photorealistic virtual avatar created through reconstruction of gaze and eye contact. Image courtesy of Schwartz et al. (2019)

Collaborative virtual environments (CVEs) allow multiple co-located or remote users to interact with each other and to collaborate on tasks. CVEs differ from other traditional group interaction or teleconferencing applications, such as video conferencing, instant messaging, and email, in that, instead of connecting users from different environments (locations), CVEs create an immersive virtual environment common to all the participants and immerse the users and task related information into that environment. This shared virtual environment provides users with a common spatial and social context for interaction and collaboration (Roberts et al. 2003). Eye tracking in collaborative virtual environments has been used to improve user representation, to aid multiparty communication and as a modality for user interaction Fig. 3.

#### 3.3.1 Representation

Users in CVEs are generally represented as virtual avatars. Natural looking and personalized avatars have been shown to improve immersion and presence in VR and to aid better communication between users Waltemate et al. (2018). Rendering the human face accurately, however, is still a particularly challenging problem. This stems from the extreme sensitivity of humans to rendering artifacts in photo-realistic facial renderings which leads to the Uncanny Valley problem (Mori et al. 2012). Previous studies show that avatars with realistic eye gazes look more natural and realistic (Garau et al. 2003); therefore, virtual avatars that have human face models should be modeled with realistic eye movements or eye movements that are consistent with human ocular behavior. To this end, eye tracking has been used to monitor users’ eye movements to allow for better reconstruction of avatars’ eyes and faces.

Different approaches have been used to create realistic artificial eyes for avatars in CVEs. The first approaches modeled the eyes as spheres or half spheres and used high resolution pictures of the human eyes as texture maps (Ruhland et al. 2014) and were not anatomically realistic. Later approaches, however, have considered the eye’s physiological features and tried to recreate them in artificial eyes. An interesting example in this area of research involves modeling changes in pupil diameter that occur as reactions to changes in light or as a function of emotional arousal (Ruhland et al. 2014). However, in order for the artificial eyes to appear realistic, they should also try to replicate the characteristic movements of the human eye. To this end, various studies have tried to animate realistic eye movements on avatars. Most of these techniques rely on eye trackers to accurately track the user’s eye movements and various models to reconstruct human eye movements based on the physiological characteristics of the eye. Ruhland et al. (2014) and Duchowski (2018) have provided an excellent review of the most widely used techniques for avatar eye movement animation.

Recent developments in deep learning have led to more efficient techniques for human face reconstruction with multimodal approaches that use eye tracking in combination with other modalities. An example application in this line of research is the recent work by Richard et al. (2020) which proposes a technique to generate photo-realistic face models using audio and eye gaze signals. In this work, the authors use codec avatars (Lombardi et al. 2018)—a class of deep models for generating photo-realistic face models that accurately represent the geometry and texture of a person in 3D that is almost indistinguishable from video. The authors develop a real-time solution for the animation of the avatars full-face geometry and texture using deep multimodal models trained on audio and gaze data. While simple face animation models may use simple reconstruction approaches, for example: eye gaze only affects eye shape and audio only affects mouth shape, multimodal approaches use multiple data streams, such as gaze and audio, to dynamically determine how these signals collectively affect each facial feature.

#### 3.3.2 Communication

According to Burgoon et al. (1994), social human communication encompasses verbal and non-verbal communication, references to objects and references to the environment. The eyes play an important role in non-verbal communication (Steptoe et al. 2010) and in providing references to objects and the environment. Deictic gestures are one of the most fundamental forms of communication that allow users to indicate objects they are referencing (Mayer et al. 2020). Although the typical deictic gesture in humans is performed through pointing by extending the arm and the index finger, previous work has shown that pointing in VR has limited accuracy (Mayer et al. 2018). As a result, eye gaze has been used as a natural way of providing deictic references (i.e., indicating where your partner is looking at) in CVEs (D’Angelo and Gergle 2016). Among others, the study by Pejsa et al. (2017) is a good example, where the authors developed a gaze model that would allow virtual agents to signal their conversational footing, i.e., signaling who in the group are the speakers, addressees, by-standers, and over-hearers.

#### 3.3.3 Interaction

In CVEs, interaction techniques are aimed at allowing the cooperative manipulation of objects or interaction elements in the VE. Cooperative manipulation (Co-manipulation) refers to the situation where two or more users interact cooperatively on the same object at the same time. Eye gaze-based interaction, among other methods, has been used in unimodal or multimodal fashion to allow CVE users to select or manipulate objects cooperatively. The recent work by Piumsomboon et al. (2017) is a sample application of eye tracking for cooperative manipulation. The authors present a collaborative system combining both VR and AR that supports bimodal eye gaze-based interaction. In this system, collaborating users could use their eye gaze to select objects and cooperatively manipulate them with their hands. The technique also allows collaborating parties to gaze at the same target object to trigger an action.

#### 3.3.4 Summary

The eyes are an important part of the face, as a result any future advancement in avatar representation in CVEs needs to be complemented with techniques that allow for precise representation and rendering of the eyes and their natural movements. Although great advancements have been made on representing users with high fidelity virtual avatars, there is still room for improvement in this area.

### 3.4 Education, training and usability

Learning is an important application of VR, and eye tracking in VR shows great promise for the assessment of learning outcomes and improving the learning process. VR has the potential to provide an immersive simulation-based training environment, where learners can hone their skills in an environment that is safe to fail and allows correction and repetition with minimal costs, while also allowing the trainer to control the learning environment down to the smallest details. As a result, VR and eye tracking has been used for education and training in several domains including transportation Lang et al. (2018), military, medicine Xie et al. (2021) and sports Bird (2020); Pastel et al. (2022).

The most common traditional approaches of evaluating learning performance in simulated learning environments include post-experiment interviews, questionnaires and think-aloud protocols (Xie et al. 2021). However, since the first two methods collect data after the experiment, the participants’ memory might fade and events might be reconstructed, potentially making the results subjective and irrelevant (Renshaw et al. 2009). Eye tracking allows the researcher to quantitatively evaluate the learner’s visual, cognitive and attentional performance during the experiment without interfering with the participant’s learning experience.

In their review of the use of eye tracking in learning, Lai et al. (2013) summarized the eye tracking indices that are most commonly used in training environments into two dimensions: types of movements (such as fixation, saccade, mixed) and scale of measurement (temporal, spatial, count). Temporal measurement indicates that eye tracking is measured in a time dimension, e.g., the time spent gazing at a particular area of interest or time to first fixation. Temporal measures aim to answer the when and how long questions in relation to cognitive processes. Spatial scale indicates that eye measurement is measured in a spatial dimension, e.g., fixation position, fixation sequence, saccade length and scanpath patterns. Spatial measures answer the where and how questions of the cognitive processes. The count scale indicates that eye movements are measured on a count or frequency basis, e.g., fixation count and probability of fixation count. Following this, Rappa et al. (2019) have identified several themes on how eye tracking has been used to aid learning and evaluate learning performance in VR, and how the scales of eye measurement summarized by Lai et al. (2013) relate to each theme. In this section, we explore applications of eye tracking in VR along these themes.

#### 3.4.1 Measuring cognitive skills

Traditionally, the interview procedure-based think-aloud protocol has been the most frequently used technique to understand cognitive processes during learning (Lai et al. 2013; Xie et al. 2021). However, as discussed above, this method could be subjective and suffers from validity issues. Eye movement tracking allows researchers to identify and measure the aspects of the learning environment that influence cognitive skill acquisition. Previous studies show that temporal characteristics of fixation, such as fixation duration, are associated with cognitive effort (Renshaw et al. 2009). According to Kiili et al. (2014), longer fixation duration might indicate increased cognitive effort suggesting that participants are engaging in analysis and problem-solving. Whereas shorter fixation duration might suggest that participants might be glossing over content because of their difficulty interpreting and comprehending it. Moreover, a review by Souchet et al. (2021) identifies that cognitive load can be measured with pupil diameter in learning tasks.

#### 3.4.2 Measuring affective skills

VR simulations have been successfully used to teach inter-personal, communicative and other affective skills. Affect refers to feelings, emotions and moods. The most common current methods of assessing affect and affective skill acquisition in simulated learning environments are self-reported measures that usually interrupt the learning experience to collect verbal or questionnaire-based responses from participants, or methods that collect data after the experiment, which may produce unreliable or biased results as discussed above (Tichon et al. 2014). Tichon et al. (2014) underscore the importance of uninterrupted and continuous measures of affect, and introduce a technique that measures the anxiety of pilot trainees in a flight simulation using eye movements and pupillometry. The results of the study indicate that fixation duration and saccade rate corresponded reliably to pilot self-reports of anxiety which suggests eye tracking-based measures could serve as reliable measures of affective skill acquisition during simulated training.

#### 3.4.3 Measuring visual attention

Visual attention refers to the ability of the human visual system to selectively process only relevant areas of interest from the visual scenes (Borji et al. 2019). Modeling users’ visual attention in VEs allows us to understand how people explore VEs and what elements of the VE attract their attention. Previous studies indicate that experts and novices show different visual attention patterns in learning tasks (Duchowski 2018). However, previous research findings have been inconsistent when looking at the average fixation duration exhibited by experts and novices. Some studies show that experts tend to exhibit shorter average fixation duration; they make better use of visual elements outside their foveal region and make use of a larger visual span area (Duchowski 2018). A meta-analytic review by Mann et al. (1998) also showed that experts used fewer fixations of longer duration compared to no experts. The review points out that experts’ eye movements are moderated by several factors including the task type and the environment. However, a study conducted outside VR by Eivazi et al. (2017) has found the opposite results showing that experts in a neurosurgery task showed longer fixation duration compared to novices. Moreover, a study by Harris et al. (2020b) has found that there is no significant differences in fixation duration between experts and novices. Although visual attention in simulated learning environments has been used to measure the expertise level of trainees and to understand visual learning strategies of experts, the inconsistency of findings shows that there is still need for more research in this area.

#### 3.4.4 Assessing learning outcomes

Eye tracking could be used to analyze learning outcomes and learning performance in VR-based training scenarios (Tichon et al. 2014). Previous studies (Rappa et al. 2019) have found that average fixation duration and number of revisits can predict the learning outcomes or the effectiveness of VR-based training. Identifying and predicting learning outcomes could allow participants to identify their areas of strengths and weaknesses in the given learning task. Additionally, a visual behavior known as the quiet eye has been shown to be a characteristic of high levels of expertise, particularly in tasks that require motor skills (Vickers 2000). Quiet eye is the final gaze fixation prior to the execution of a movement towards a target. However, in a study of quiet eye in virtual reality, Harris et al. (2021b) found that the quiet eye had little to no impact in skill execution.

#### 3.4.5 Measuring immersion

Immersion refers to the objective level of sensory fidelity provided by a VR system, whereas presence is defined as the subjective sensation of being there in a scene depicted by a medium, usually virtual in nature (Bowman and McMahan 2007; Barfield et al. 1995). In simulated learning environments, it is widely believed that better immersion leads to better learning outcomes (Jensen and Konradsen 2018). Previous studies show that eye movements correlate with the immersiveness of virtual environments and could be used to measure immersion in virtual environments (Jensen and Konradsen 2018). Results from a study by Jennett et al. (2008) indicate that the participants’ number of fixations per second significantly decreased overtime in an immersive condition and significantly increased overtime in a non-immersive condition. These results indicate that participants were more concentrated in the immersive condition, while they were more distracted in the non-immersive condition.

#### 3.4.6 Measuring usability

Usability is often associated with ease of use and is an important part of user experience. Usability deals with measuring the aspects of the virtual environment that make it easy to use, understandable and engaging (Kiili et al. 2014). The design aspects of the environment and the elements within it could affect the cognitive performance of the participant (Kiili et al. 2014). Thus, simulated learning environments should be designed with optimal task performance in mind. Eye tracking could help in identifying factors that affect usability and be an objective measure of usability in VEs (Kiili et al. 2014). In particular, average fixation duration and gaze points within areas of interest indicate which elements of the scene attract the user’s attention and are correlated with virtual environment design features (Renshaw et al. 2009).

#### 3.4.7 Summary

Eye tracking in VR has been used to assess learning performance in simulated learning environments. Care should be taken, however, in translating the eye movement-based performance measurements. Kiili et al. (2014) remind that eye tracking data reflect what the user perceives, but do not tell whether or not the user comprehends the information they were presented in the experiment. Developing accurate metrics that could measure knowledge comprehension based on eye tracking data is an open area for future research. On the other hand, the degree to which skills gained in simulated environments are transferable to the real world needs further research (Harris et al. 2021a). Moreover, there are concerns that differences between the actual task and the simulated training task, and the limited interaction and realism provided by VR could be detrimental to VR-based training programs (Ruthenbeck and Reynolds 2015; Harris et al. 2020a). More research is needed in this area to develop better eye tracking data interpretation methods to measure learning outcomes; to ensure that VR-based training tasks are effective; and to ensure that the skills learned in simulated environments can be transferred to the real world.

### 3.5 Security and privacy

As the adoption of VR expands, the number and variety of VR applications has been growing steadily. Many of these applications require users to authenticate themselves or to enter their personal details—tasks that require a high level of security. With the current surge in the integration of eye trackers in HMDs, eye movement data could be used to implement robust and non-intrusive authentication methods. Personal authentication and recognition techniques based on eye movements (not to be confused with iris recognition) have been used successfully for several years (Katsini et al. 2020). Due to differences in gaze behavior and oculomotor physiology, there are certain eye movement characteristics that are unique for each individual. This difference can be exploited to implement a biometric identification system.

A user’s patterns of eye movements depend on the user’s distinct characteristics and are very different among users. Visual stimuli produce varied eye movement behaviors and patterns among users. Pupil size, saccade velocity and acceleration, fixation behavior and pupil center coordinate and its displacement between consecutive samples have been identified as dynamic eye movement features that are specific to individual users and suitable to be utilized for authentication (Kinnunen et al. 2010; Eberz et al. 2015; Liang et al. 2012). These eye movement metrics can be measured by most VR-based eye trackers and can be fed into a decision or classification algorithm to develop an eye movement-based authentication system.

Additionally, due to the fact that users’ eyes are totally obstructed from outsiders when using HMDs and the idiosyncratic characteristics of eye movements, using eye movement-based authentication could be a spoof proof authentication method. Eye gaze data can be employed to enable explicit or implicit authentication of VR users. When eye gaze-based authentication is used in an explicit fashion the user has to first define a password that involves consciously performing certain eye movements, then the user is authenticated by recalling these movements and providing them as input. Mathis et al. (2020) presented an authentication method where VR users can be authenticated by selecting digits from a 3D virtual cube using their eye gaze, head pose or hand controller. The authors report that while the eye gaze-based method was slightly slower than when using the two other input techniques, it was highly resilient to observation-based attacks with 100% of observation-based attacks being unsuccessful.

Implicit eye gaze-based authentication refers to authentication methods that aim to verify the identity of users implicitly without requiring the user to remember a secret. Implicit authentication is based on the user’s unconscious eye movements and can be performed continuously in the background. Various studies have explored implicit eye movement-based biometrics in VR (Zhang et al. 2018; Iskander et al. 2019; Liebers and Schneegass 2020; Pfeuffer et al. 2019; Lohr et al. 2018). The main advantage of implicit authentication methods over explicit authentication methods is the fact that they can be performed in the background without interrupting the user and can be performed continuously throughout a session to ensure intruders do not access already authenticated sessions. Studies show that implicit eye gaze-based authentication methods have comparable or better accuracy than explicit authentication methods, with some studies reporting that 86.7% accuracy can be achieved with only 90 seconds of data (Zhang et al. 2018).

#### 3.5.1 Summary

Eye movement data holds great potential to be used for explicit or implicit identification of VR users without interrupting their virtual experience. On top of developing better algorithms for implicit eye movement-based authentication, future work in this area could include the development of intrusion-proof transmission and storage techniques or protocols for eye tracking data that take into consideration the highly sensitive nature of the data. Moreover, the development of eye tracking data privacy practices and policies that outline and restrict the use of the data only for necessary purposes is an important area for future research.

### 3.6 Marketing and consumer experience (CX) research

Eye tracking can help us to better understand consumer’s visual attention and provides rich information about the cognitive and emotional state of consumers (Lohse 1997). Most of the current research on consumer behavior that involves collection of eye tracking data is conducted in laboratory settings, with a majority of studies using 2-D pictures and desktop eye trackers, however the behavior observed in the laboratory may be different from what is observed in the field (Kahn 2017). Therefore, various studies suggest that consumer research should be done in the real world or at the point of sale (Meißner et al. 2019). However, conducting research in the real world—in the field—is cumbersome and could introduce uncontrolled factors to the experiment which in turn could make the experiment hard to replicate. Several reviews (Bigné et al. 2016; Wedel et al. 2020; Alcañiz et al. 2019; Loureiro et al. 2019) provide an overview of the various advantages of using VR for customer research. VR allows the experimenter to control every detail of the experiment similar to experiments conducted in laboratory settings, while providing an immersive virtual environment that feels like reality and provides the customer with freedom of movement similar to a real-world shopping experience. As a result, eye tracking in VR is a powerful tool for CX research. Lemon and Verhoef (2016) conceptualized CX as a customer’s journey over time during the purchase cycle, and divided the purchase journey into three stages: the pre-purchase stage, the purchase stage and the post-purchase stage.

*The pre-purchase stage* encompasses the CX from when the customer recognizes the need to purchase a product up to the time the need is satisfied with a purchase. Eye tracking is widely used in the pre-purchase stage. This stage includes various marketing practices such as advertising, design of product attributes such as package design and testing new products.

Eye tracking plays an important role in understanding the effectiveness of advertisements. Advertisement has been a key application area for VR and augmented reality (AR) in marketing. In 2017, $167 million was spent on VR and AR advertising alone, and this figure is expected to grow to 2.6 billion in 2022. This growth is in part driven by recent findings that show that VR and AR ads have higher engagement and click through rate than traditional advertising methods (Grudzewski et al. 2018; Van Kerrebroeck et al. 2017). For example, Hackl and Wolfe (2017) mention that marketing campaigns that use AR have an average dwell time of 75 s, compared to an average dwell time of just 2.5 s for traditional radio and TV ads, and that 71% of shoppers would shop at a retailer more often if they used AR. Wang et al. (2019) have proposed a system for the analysis of dynamic user behavior when watching VR advertisements. The experiment used eye tracking data to measure the participants’ visual behavior around the virtual ads.

**Fig. 4:**
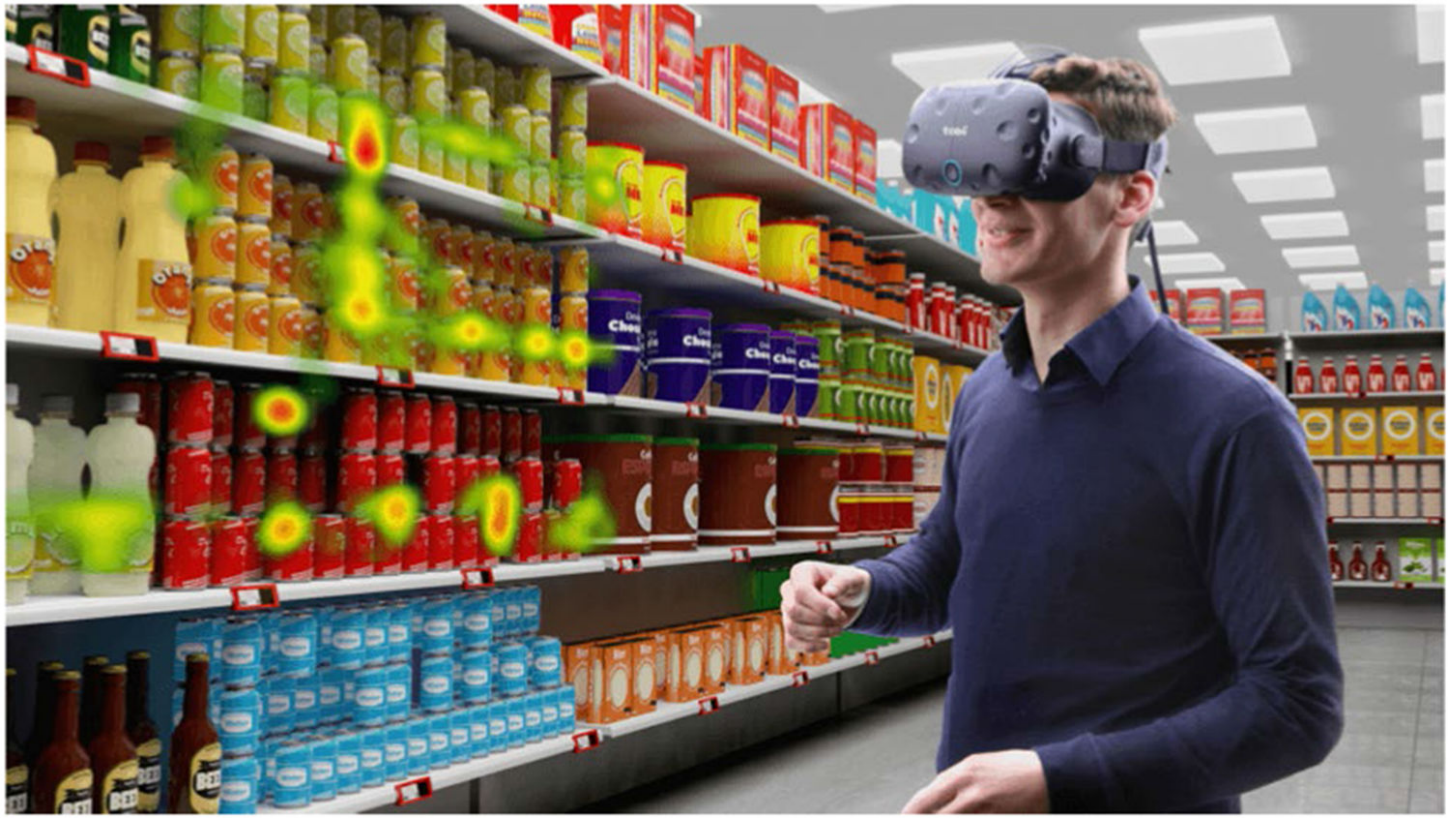
A combined image showing which items in a virtual store attract the user’s visual attention. Image courtesy of Tobii AB

Product attributes, such as its package’s design, play an important role in marketing and influencing the consumer’s purchase choice. Creusen and Schoormans (2005) identified that product appearance plays an important role, which includes drawing the consumer’s attention among other roles. Various studies have shown that consumers choose the products that attract their visual attention (Bigné et al. 2016). Visual attention (see section 3.4) can effectively be measured using eye tracking. Most studies on product appearance are conducted in laboratory settings which fail to capture the visual context in which the product will be situated. Product appearance studies in the real world, on the other hand, fail to control all experiment variables which could greatly affect experiment results. VR may help researchers to close this gap by allowing them to rapidly develop new product prototypes and place them in immersive environments while giving the experimenter the ability to control all aspects of the experiment environment. A particularly good example is a recent study conducted by Meißner et al. (2019) that combines VR with eye tracking to provide a naturalistic environment for research on package design. Rojas et al. (2015) conducted a study in which the consumer perception of a virtual product with photographic representation of the same product. Eye tracking data were used to analyze the participants’ gaze behavior during the experiment Fig. 4.

*The purchase stage* includes all customer interactions with the brand and its environment during the purchase event itself. At this stage, eye tracking is mainly used to examine the user’s behavior to understand how they process the information at the point of sale so we can get insights into their product choice, customer experience, and shopping behavior. This insight could then be used to drive the user towards new purchases (e.g., recommender systems) and to customize the shopping experience. Example applications of eye tracking and VR at this stage include a study by Bigné et al. (2016) where the authors developed a system that uses data from eye movements, and a VR-based virtual shopping experience, to track consumer behavior and report consumer paths, seeking behavior, purchase behavior, and the time a person spends on each task. Pfeiffer et al. (2020) demonstrates that eye tracking can be used at the point of sale to tailor recommender systems to individual customers’ shopping motives. They conducted two experiments where one was conducted in VR and the other was conducted in a physical store. The results of the experiment indicate that the information search behavior of users in VR might be similar to the one used in the physical store.

*The post-purchase stage* encompasses customer interactions with the brand and its environment following the actual purchase. Although there is an increasing number of VR applications in this stage, we could not find applications that use both VR and eye tracking.

#### 3.6.1 Summary

Eye tracking coupled with VR provides a powerful tool that can help advance marketing and CX research in various areas including new product development, advertising, and assessment of consumer behavior and engagement. The applications of VR with eye tracking are under-utilized at the post-purchase stage, where there are potential promising applications that could help companies to analyze consumer behavior and interactions with the purchased product or service.

### 3.7 Clinical applications

Clinical applications of eye tracking in VR mainly include diagnostics and assessment, therapeutic uses and interactive uses in a clinical context.

#### 3.7.1 Diagnostic applications

The potential of using eye movements to identify markers of psychiatric, neurological and ophthalmic disorders is well researched (Trillenberg et al. 2004; Clark et al. 2019). Because different eye movement types are controlled by different brain regions and different neural circuits, examination of the kinematics of various types of eye movements can provide clues to disorders in the underlying neural structures (Trillenberg et al. 2004; Tao et al. 2020). Targeted VR applications that mimic diagnostic tasks used by clinicians can be used to elicit eye movement abnormalities associated with psychiatric and neurological disorders. Eye tracked VR, thus, can be used to develop complete diagnostic tools for psychiatric, neurological and ophthalmic diseases.

Eye tracking in VR has shown great potential in the diagnosis of neurodegenerative conditions such as Parkinson’s disease (PD) and Alzheimer’s disease (AD). The diagnosis of neurodegenerative disease is usually made by physicians based on visible signs and symptoms. However, these signs and symptoms may take years to develop. Previous studies show that early stages of PD and AD can be detected through the observation of eye movement abnormalities. For example, Orlosky et al. (2017) developed a VR and eye tracking-based system to diagnose neurodegenerative disease and evaluated the system by conducting experiments on patients with Parkinson’s disease. The main focus of the system is to evoke eye movement abnormalities using virtual tasks in VR associated with neurodegenerative disease so that a correct diagnosis can be made by observing these abnormalities.

Furthermore, VR with eye tracking has been employed to develop diagnostic tools for various ophthalmic diseases and disorders. Tatiyosyan et al. (2020) used a VR headset with an eye tracker to develop an optokinetic nystagmus (OKN)-based tool to test contrast sensitivity. Measuring contrast sensitivity in low vision patients is used to determine the stage of visual impairment. Miao et al. (2020) used a VR headset with integrated eye tracking to develop an automated test for ocular deviation for the diagnosis of strabismus in patients. Current diagnosis of strabismus uses cover tests that rely on the doctor’s experience, which could be susceptible to human error.

#### 3.7.2 Therapeutic applications

In the clinical context, eye tracking in VR has been used for neuropsychological, physical and ophthalmic therapeutic interventions (Lutz et al. 2017). In therapeutic applications, eye tracking is commonly used as an objective measure of the patient’s symptoms. The objective metric could then be used to assess the progress of treatment or to personalize the treatment experience to the patient.

Eye tracking has been used as an objective metric in the VR-based treatment of Generalized anxiety disorder (GAD) and various phobic disorders. GAD is a mental health condition marked by excessive, exaggerated and consistent anxiety and worry about everyday life events. Phobias are similar to anxiety disorders, but the anxiety is specific to an object or situation. The treatments for those disorders commonly require patients to confront the situations they fear through a process known as exposure therapy. Although exposure therapy has been proven to be highly effective, recreating the feared situations in real life is challenging and could put the patient in danger, for example, exposing a person who has fear of heights in an actual elevated space. VR, on the other hand, can provide a safe simulated environment for exposure therapy. Various studies have demonstrated that persons with psychological disorders show attentional biases and different eye movement patterns when they are exposed to the situation they fear. Consequently, eye tracking has been an integral part of virtual reality exposure therapy (VRET). For example, in a VR-based therapy for social phobia, Grillon et al. (2006) used eye tracking movement tracking to objectively assess eye gaze avoidance, a persistent symptom in social phobia.

VR-based applications have a demonstrated potential for treatment of various psychiatric and neurological disorders. However, the treatment tasks should be managed effectively to keep the user engaged and to ensure that the task is within the user’s capability. Bian et al. (2019) discusses that a task that is too difficult for the patient could be overwhelming and cause anxiety while a task that does not fully utilize the patient’s capability might cause boredom. Eye tracking in VR can be used to provide real-time data to assess the performance and engagement of patients and create a feedback loop to dynamically update the treatment experience in response to the user’s engagement and performance.

#### 3.7.3 Interactive applications in a clinical context

Various clinical applications of VR require the user to interact with the environment. Many patients, however, might not have the physical ability (e.g., patients with motor disabilities) to use a hand-held VR control device. Previous studies have shown the suitability of eye tracking-based interaction interfaces for clinical VR applications. Eye tracking-based interaction is mainly used to increase the immersiveness of the VR experience to illicit a stronger sense of virtual presence in patients.

Al-Ghamdi et al. (2020) evaluated the effectiveness of VR-based therapy at reducing the pain experienced by patients with severe burn wounds during non-surgical wound debridement procedures. However, the injuries prevent most patients from using conventional VR controllers to interact with the virtual environment. Thus, they investigated whether eye tracking-based interaction can enhance the analgesic effectiveness of the VR-based distraction for pain management. The results of the study indicate that interactive eye tracking improved the immersiveness of the virtual environment and as a result increased how effectively VR reduced worst pain during a brief thermal pain stimulus. Another study by Pulay (2015) proposed the use of interactive eye tracking to motivate children with severe physical disabilities (like Tetraparesis spastica) to take an active role in their VR-based rehabilitation programs.

#### 3.7.4 Summary

Eye tracking in VR in the clinical context is used for diagnostic, therapeutic, and interactive purposes. Neuro-ophthalmic diagnosis is traditionally conducted in a very rudimentary manner at the patient’s bedside. Development of uniform HMD-based diagnostic tools with precise stimulus control to elicit specific and relevant eye movements, e.g., pursuit, saccades, nystagmus, etc., along with automated analysis of the resulting eye movements holds great promise.

Clinical applications could also benefit from the development of more usable applications that are easy to comprehend and use for the patient as well as the clinician practitioner. This has the potential to allow patients to self-diagnose or self-treat neurological disease and provide clinicians with easy-to-use tools.

Most current clinical applications of VR and eye tracking use consumer hardware that may not be appropriate for clinical use. For example, Lutz et al. (2017) mention that most HMDs have to be modified by removing, enclosing or replacing their textile foam and Velcro components in order to comply with clinical hygiene regulations. Most HMDs and their eye tracking components also cannot withstand clinical disinfection procedures. Thus, there is still work to be done to produce clinical grade HMDs.

In conclusion, as the data quality of eye trackers, and the VR hardware and software continue to improve, we expect the clinical applications of eye tracked VR to continue to grow.

## 4 Challenges and limitations

In this section, we discuss the inherent challenges and limitations of eye tracking and how these challenges affect eye tracking in VR.

### 4.1 Technological limitations

#### 4.1.1 Eye tracking data quality

Issues with eye tracker data quality are arguably the biggest technological challenges for eye tracking in VR. Table 1 shows the manufacturer reported data quality specifications for the currently most used HMD-based eye trackers.

##### Spatial Precision:

Applications that use small fixational eye movements, like tremors, drifts and microsaccades require high-precision eye trackers. Andersson et al. (2010) point out that for such tasks the eye tracker should have an RMS precision value that is lower than 0.03°. Table 1 shows that not all manufacturers of VR-based eye trackers report the precision value of the eye trackers, and those that have reported precision values seem to have precision values that are worse than the recommended range needed to detect small fixational movements.

##### Spatial Accuracy:

Table 1 shows that the most popular HMD-based eye trackers report accuracy values between 0.5° and 1.1°. However, manufacturer reported specifications could be misleading, as these metrics are often measured under ideal conditions and do not reflect the accuracy under realistic usage scenarios (Adhanom et al. 2020a).

Additionally, most current HMD-based eye trackers have the highest accuracy and precision in a small central region of the FOV. Outside this region, accuracy and precision drop substantially. For example, the HTC Vive Pro Eye reports accuracy values of 0.5° - 1.1° within the central 20° of the FOV. Outside this region, the accuracy is not guaranteed. This limits researchers or developers from using the whole FOV for eye tracking-based experiences.

Regardless of the application, performance across all categories would benefit from improvements in the precision and accuracy of eye tracking and also from improvements in identification and labeling of eye movement types. Advances in both of these areas can be achieved by leveraging additional sources of information beyond the eye images that underlie VOG. Simultaneous use of eye data and scene information can help constrain eye position estimates based, for example, on the assumption that users are much more likely to look at an object than the empty space next to an object (Tafaj et al. 2012). Similarly, simultaneous analysis of both head and eye movement data allows reconstructing gaze in world coordinates, which allows identifying eye movement types that would be difficult to identify based on eye movement alone (Hausamann et al. 2020; Kothari et al. 2020). As with most current information processing challenges, application of machine learning techniques has shown promise in advancing eye tracking precision, accuracy, as well as identification of eye movement types (Kothari et al. 2020; Yiu et al. 2019).

##### Latency:

High latency could cause elements of the stimulus that are supposed to respond to eye movement to lag behind the eye movements. When this lag is big enough that it is perceptible by the user, it could severely affect the virtual experience. This could be particularly problematic in interactive applications or GCDs which change some part of the stimulus in real time in response to eye movements. Albert et al. (2017) points out that foveated rendering is tolerant to latencies of about 50-70 ms. For latencies beyond this range, foveated rendering could be perceptible to the user and lose its effectiveness. Although latencies have greatly decreased in current HMD-based eye trackers, a recent study analyzed the latency for most of the currently available HMD-based eye trackers and reported that the latencies for the mostly used HMD-based eye trackers ranged from 45 ms - 81 ms (Stein et al. 2021). Although these latencies do not cause significant issues for non-real-time applications, they could hamper real-time use of eye tracking data in VR.

##### Sampling Rate:

As discussed above, the precise measurement of small amplitude eye movements such as fixational eye movements and low amplitude saccades requires eye trackers with high sampling rates. In Sect. 2.3.1, we have discussed that the sampling rate should be at least twice the speed of the eye movement to be recorded. Table 1 shows that current HMD-based eye trackers have sampling rates that fall in the range 100-200 Hz. These relatively low sampling rates show that most of the commonly used HMD-based eye trackers are not well suited to accurately record low amplitude eye movements.

#### 4.1.2 Calibration

The time consuming and repetitive nature of the calibration procedure could be an obstacle for the wide adoption of eye tracking and could make eye tracking unattractive for applications that require instant use.

Moreover, some users, such as children and users with attentional deficits, have difficulty completing the calibration procedure as they lose interest in the procedure after a few targets have been shown resulting in an unsuccessful calibration procedure (Blignaut 2017). Alternative eye tracking procedures have been explored to address the issues with the calibration procedure, the most common of which use smooth pursuit eye movements to dynamically calibrate the eye tracker without explicitly asking the user to look at point targets (Blignaut 2017; Drewes et al. 2019). Although these methods require less time and can be performed without the user being aware of the calibration procedure, they generally produce lower quality eye tracking data. As a result, these calibration methods are not in use in any HMD-based eye tracker we are aware of (Drewes et al. 2019).

After the calibration procedure, changes in lighting, eye geometry and the relative position of the eye tracking camera with respect to the user’s eyes could cause calibration errors. The latter is the main cause of calibration error in HMD-based eye trackers, as small movements of the VR headset due to the user’s movements could cause calibration errors. All these factors together cause the calibration to decay—the calibration error and spatial accuracy of the eye tracker worsens over time. We call this decay *drift*. Drift is a common cause of low quality eye tracking with some eye trackers showing calibration drift of about 30% in the first 4 minutes and 30 seconds after calibration (Ehinger et al. 2019). Calibration errors are hard to deal with due to their dynamic nature. However, the severity of calibration errors could be reduced by making sure the HMD does not move relative to the head after calibration and repeating the calibration procedure multiple times during long sessions. Further research is still needed to develop calibration procedures that are easy, comfortable, and robust to drift.

### 4.2 Data privacy and security challenges

With eye tracking becoming ubiquitous in new HMDs, there has been growing concern about the privacy of eye tracking data collected on these devices. Kröger et al. (2020) and Steil et al. (2019) explained that eye tracking data contain rich information content that could be used to infer a vast amount of personal information about the user including: the user’s interest in a scene; the user’s cognitive load and cognitive state; various mental disorders including Alzheimer’s, Parkinson’s and schizophrenia; the user’s personality traits; and other sensitive data including the user’s gender, age, ethnicity, body weight, drug consumption habits, emotional state, skills and abilities, fears, interests, and sexual preferences (Kröger et al. 2020). Adams et al. (2019) point out that a majority of VR developers do not follow proper privacy practices to guard user data collected in VR systems. They also mention data collected from users in VR systems could be collected or transferred to third parties without the user’s knowledge or be leaked through known security vulnerabilities.

The near infra-red cameras used in HMD eye trackers collect tens or hundreds of high-quality images of the eye per second. The susceptibility of these images to attacks is a major concern. John et al. (2019,2020) point out that these images contain iris-patterns of the user and if an intruder gets access to even a single image from this data stream, they have effectively captured a gold standard biometric—iris authentication. John et al. (2020) introduces a hardware-based technique to degrade the images collected by the eye tracker so that the images cannot be used for iris authentication while still allowing the utility of gaze tracking. The results of the system indicate that the average Correct Recognition Rate—the rate at which users’ can be identified from their eye images—was reduced from 79% to 7% when using the system.

Given the amount of sensitive information that can be gathered from eye tracking data and the lax privacy practices in the current VR ecosystem, Steil et al. (2019) explains how eye movements recorded using HMD-based eye trackers could be a potential threat to users’ privacy. They point out that there is an urgent need to develop privacy-aware eye tracking systems—systems that provide a formal guarantee to protect the privacy of their users. They also propose a method that could be used to protect users’ privacy in eye tracking based on differential privacy—a method that adds noise to eye tracking data to hide the privacy-sensitive information in the data while still allowing the data to be used for the desired task.

Despite these findings, there is a need for more research in privacy and security as it relates to eye tracking in VR. The rapid technological advancement in VR hardware and the increased integration of eye tracking in HMDs have resulted in a surge of customer-facing applications that use eye tracking data. Therefore, it is important for researchers and practitioners to develop tools and standards that preserve the user’s privacy and enhance the security of eye tracking data.

### 4.3 Safety issues

Current customer-facing HMD-based eye trackers are VOG-based eye trackers that use infra-red (NIR) radiation to enhance the contrast between the pupil and the iris. Most eye trackers use near IR (NIR) light sources with wavelengths around 880 nm which are invisible to the human eye. However, previous studies have shown that prolonged exposure to IR could have a damaging effect on the user’s eyes (Kourkoumelis and Tzaphlidou 2011). The proximity of the IR source, the length of exposure and the number of IR sources have been identified as factors that could potentially increase damage to parts of the eye including the cornea, the lens and the retina (Kourkoumelis and Tzaphlidou 2011). Considering that eye trackers in VR HMDs use multiple IR sources in close proximity to the eye, and that VR applications are designed for prolonged use, the potential longterm hazards of IR radiation from HMD-based eye trackers should be thoroughly investigated.

## 5 Conclusion

It is clear based on the many applications mentioned above that eye tracking will soon become an integral part of many, perhaps most, HMD systems. We therefore expect research and development surrounding eye tracking in HMDs to accelerate and expand in the coming years.

Eye tracking in VR has shown great potential to improve rendering efficiency in VR systems and to help enable more comfortable and immersive VR experiences. The most prominent example in this area is gaze-contingent (foveated) rendering. However, more research is needed in this area to understand the perceptual effects of gaze-contingent rendering. More work also needs to be done to reduce the end-to-end latency of eye tracking systems in VR to make gaze-contingent changes in the environments imperceptible to end users.

Moreover, as the user base in VR grows and the need for developing accessible interaction techniques increases, eye tracking has been explored as powerful modality to develop accessible and hands-free interaction techniques for VR systems. Eye tracking could be explored to enable individuals with severe motor impairments to interact with VR systems. There are still challenges with using eye tracking for precise interaction, and more work could be done to enable multimodal or semi-autonomous interaction techniques that solve the challenges with eye tracking-based interaction. Moreover, with more and more sensors being embedded in VR HMDs, sensor fusion (i.e., combining eye tracking with other sensors) is another area for future research that holds promise to further increase the accuracy of eye tracking-based interaction.

VR is used for developing learning and skills training applications as it provides trainees an immersive environment that is safe to fail and allows repetition and correction with minimal cost. Eye tracking in VR has been used for the assessment of learning outcomes and improving the learning process. More research is needed in this area to develop better eye tracking data interpretation methods to measure learning outcomes; to ensure that VR-based training tasks are effective; and to ensure that the skills learned in simulated environments and can be transferred to the real task.

Eye tracking could supplement VR to provide powerful tools that can help advance marketing and CX research in various areas including new product development, advertising, and assessment of consumer behavior and engagement. Previous research has mostly focused at the pre-purchase and purchase stages of the customer journey. However, there is potential for interesting future research on the post-purchase stage that could help companies to analyze consumer behavior and interactions with the purchased product or service.

Eye tracking has been used in the clinical context for diagnostic, therapeutic and interactive uses. However, there is still need for further research to develop uniform and more usable clinical applications that are easy to comprehend and use for the patient as well as the clinician practitioner. This has the potential to allow patients to self-diagnose or self-treat neurological disease and provide clinicians with easy to use tools. There is also need for the development of VR and eye tracking hardware.

Additionally, eye tracking data can be used to easily identify and authenticate users in an implicit way without interrupting the user’s main task. On the other hand, this makes eye tracking data vulnerable and sensitive to data privacy and security challenges. Therefore, more work needs to be done to develop tools and standards that preserve users’ privacy and strengthen security of eye tracking systems.

In summary, eye tracking, i.e., the ability to precisely and accurately measure the user’s eye gaze, has potential to become a standard feature on consumer VR headsets. The availability of high-precision, low-latency and low-cost eye trackers in VR HMDs has led to the emergence of a host of new applications spanning multiple disciplines. This paper provides a broad review of these applications and highlights some areas for future research.

## Figures and Tables

**Table 1:** Current widely used HMD-based eye trackers and their manufacturer specifications. Note that the manufacturer specifications are calculated under ideal conditions and may be hard to reproduce in realistic settings

Device	Accuracy	Precision	Sampling rate	Latency
Fove-0	1.15°	Not reported	120 Hz	Not reported
HTC Vive Pro Eye	0.5° - 1.1°	Not reported	120 Hz	Not reported
Pico Neo 3 Pro Eye	Sub-degree gaze accuracy	Not reported	60/90 Hz	Not reported
Pupil Labs	1°	0.08°	200 Hz	8.5 ms Camera latency and 3-4 ms processing latency on i5 CPU
Varjo VR-1	1°	0.2°	100 Hz (default) or 200 Hz	20-30ms
Varjo VR-3	Sub-degree accuracy	Not reported	200 Hz	Not reported
